# Effect of S-Methyl-L-Cysteine on Oxidative Stress, Inflammation and Insulin Resistance in Male Wistar Rats Fed with High Fructose Diet

**Published:** 2015-01

**Authors:** Sithara Thomas, Gandhipuram Periyasamy Senthilkumar, Kuppuswamy Sivaraman, Zachariah Bobby, Sankar Paneerselvam, Kotten Thazhath Harichandrakumar

**Affiliations:** 1 Department of Biochemistry, Jawaharlal Institute of Postgraduate Medical Education and Research (JIPMER), Puducherry, India;; 2 Department of Medical Biometrics and Informatics, Jawaharlal Institute of Postgraduate Medical Education and Research (JIPMER), Puducherry, India

**Keywords:** Fructose, Insulin resistance, Inflammation, Oxidative stress

## Abstract

**Background:**

S-methyl cysteine (SMC) is a hydrophilic cysteine-containing compound naturally found in garlic and onion. The purpose of the present study was to investigate the protective effect of SMC on oxidative stress, inflammation and insulin resistance in an experiment of metabolic syndrome.

**Methods:**

Male Wistar rats were divided into five groups (6 rats in each group), namely; control, control+S-methyl cysteine (SMC), high fructose diet (HFD), HFD+SMC and HFD+metformin. The 60% fructose used for 8 weeks and SMC in the dose of 100 mg/kg bw/day/rat was used in the study. The fasting glucose, insulin, insulin resistance, and tumor necrosis factor alpha and erythrocyte enzymatic antioxidants were measured.

**Results:**

Increased levels of plasma glucose, insulin, malondialdehyde, tumor necrosis factor-alpha, and insulin resistance and decreased levels of glutathione, glutathione peroxidase, and catalase were found in rats on a high fructose diet. Oral administration of SMC (100 mg/kg bw/day/rat) for 60 days resulted in significant attenuation of plasma glucose, insulin, tumor necrosis factor–alpha, insulin resistance and improved antioxidant enzyme activities.

**Conclusion:**

Oral treatment of SMC is effective in improving insulin resistance while attenuating metabolic syndrome, inflammation, and oxidative stress in male rats fed with fructose rich diet.

## Introduction

The metabolic syndrome characterized by insulin resistance, hypertriglyceridemia, and hypertension is associated with increased risk of type 2 diabetes and coronary heart disease, resulting in reduced quality of life and increased risk of mortality and morbidity. The prevalence of metabolic syndrome and its interrelated complications has increased worldwide due to the modern lifestyle and an increased consumption of high-sugar diets, especially fructose.^1^ Recent findings support that increased consumption of fructose is an important contributor to the metabolic syndrome, typically resulting in insulin resistance, hypertension, and hypertriacylglycerolaemia.^2^

A number of oxidized compounds are produced during the attack of free radicals against membrane damage such as lipoproteins, proteins, and polyunsaturated fatty acids. One of them is malondialdehyde (MDA) which can be used as an indicator of oxidative stress, since its concentration in the blood increases as the result of free-radical mediated processes.^3^ The antioxidative system enables transformation of reactive oxygen species (ROS) into inactive and harmless compounds. Natural antioxidant enzymes like superoxide dismutase (SOD), glutathione peroxidase (GPx) and catalase (CAT) provide the primary defense against reactive oxygen species. Superoxide dismutase can selectively scavenge a superoxide radical by catalyzing its dismutation to hydrogen peroxide and molecular oxygen, while GPx and CAT decompose hydrogen peroxide to the unreactive species. Several studies have documented that an increase in the catabolism of fructose can be associated with the cellular energy depletion that can increase the susceptibility of cells to lipid peroxidation.^4^ Furthermore, it has been postulated that increased catabolism of fructose can accelerate free radical production similar to glucose and impairs the free radical defense system leading to oxidative stress.^5, 6^

Plants have always been considered as an ideal source of drugs and many of the currently available medicinal drugs were derived directly or indirectly from plant sources. Wide arrays of plant derived active principles have demonstrated activities consistent with their potential use in the treatment of diabetes mellitus.^7^ Garlic (*Allium sativum, *Liliaceae) is a rich source of bioactive compounds and is used in folk medicine for the treatment of various diseases.^8^ S-methyl-L-cysteine is a sulfur containing amino acid, present in garlic, and has been reported to have anti-lipidemic activity.^9^ Although numerous compounds derived from garlic have been investigated in the last 50 years to determine the benefits of garlic for human health, there are no scientific literatures available on the efficacy of SMC in high fructose fed induced hyperglycemic rats.

Hence, the present study was conducted in order to find the therapeutic potential of SMC as oral hypoglycemic and antioxidant agent. The effects produced by these treatments are compared with the standard drug, metformin. 

## Materials and Methods

*Chemicals*

S-methyl cysteine, thiobarbituric acid, and reduced glutathione (GSH) were procured from Sigma Chemicals Co. (St. Louis, MO, USA). All other chemicals were of analytical grade and were obtained from standard commercial suppliers.

*Animals*

Male albino Wistar rats at the age of five months old, weighing between 200 to 250 g (supplied by animal house, Jawaharlal Institute of Postgraduate Medical Education and Research (JIPMER), Puducherry, India) were housed in polypropylene cages with stainless steel grill. The animals were maintained at standard conditions with a 12-/12-hour light/dark cycle and all experiments were conducted in the Department of Biochemistry, JIPMER. Animal procedures were approved by the Institutional Animal Ethics Committee (Jip/Micro/Jiaec/2012 date 28.3.2012). The experiment was carried out on male rats at the age of five months with six rats in each group (according to the institute policy, six rats in each group was approved for postgraduate medical dissertation) and followed for a further two months with different treatments as given below:

Group 1: Control rats received standard rat chow.
Group 2: Control-s-methyl-L-cysteine (100 mg/kg bw/day/rat) in aqueous solution orally for 60 days.
Group 3: High Fructose Diet (HFD) rats were given 60% fructose mixed with standard rat chow.
Group 4: HFD+s-methyl-L-cysteine (100 mg/kg bw/day/rat) in aqueous solution orally for 60 days.^9^
Group 5: HFD+metformin (50 mg/kg bw/day/rat) in aqueous solution orally for 60 days.^10^

*Biochemical Analysis*

At the end of the experimental period, the rats were kept fasting for 15 hours before blood was collected from the retro orbital sinus vein. The plasma glucose was estimated using the glucose oxidase-peroxidase method in a fully automated clinical chemistry analyzer (AU-400) and the remaining samples were stored at -80ºC for the estimation of TNF-alpha (Diaclone, France) and insulin by ELISA method (Crystal Chem. Inc., USA) according to the manufacturer’s instructions. The homeostasis model insulin resistance (HOMA-IR) was calculated using the formula [fasting glucose (mmol/L)×fasting insulin (IU/mL)]/22.5.

The plasma malondialdehyde were estimated by TBARS method,^11^ The erythrocytes were prepared as described by Biswas et al.^12^ and erythrocyte-reduced glutathione content was determined using Ellman’s reagent (5,5’-Dithio-bis-2-nitrobenzoic acid) as described by Beutle et al.^13^ The whole blood hemoglobin was measured by the method of Drabkin and catalase by the method of Aebi.^14^^,^^15^

*Statistical Analysis*

All values were expressed as mean±SD. Differences between the groups were assessed by using one-way analysis of variance with (ANOVA) with post hoc analysis for pairwise comparison of the HFD group with control, SMC and HFD+metformin groups. All statistical analysis was carried out at 5% level of significance using SPSS version 19.0. 

## Results

The details on the analysis of the effect of SMC on the plasma glucose, insulin, and HOMA-IR between the study groups are given in table 1. It shows that the level of plasma glucose, insulin and HOMA-IR levels were significantly different (P<0.05) on overall between the groups. The pairwise analysis shows that the level of parameters in HFD-induced diabetic group of rats were significantly (P<0.05) higher when compared with the control group, SMC and HFD+Metformin groups. The oral treatment of SMC as well as metformin significantly decreased the glucose, insulin, and insulin resistance when compared with the HFD group of rats.

**Table 1:** Comparison of the levels of plasma glucose, insulin, and HOMA-IR between the groups

**Groups**	**Fasting plasma glucose (mg/dl)**	**Plasma insulin (ng/ml)**	**HOMA-IR**
Control	106.3±11.54	1.2±0.55	8.0±3.60
Control+SMC	91.8±7.88	1.0±0.97	6.0±6.35
HFD	149.7±12.91a,b,c	4.1±0.84a,b,c	38.2±6.01a,b,c
HFD+SMC	107±18.59	2.6±1.2	15.3±7.32
HFD+metformin	92±14.71	2.2±0.51	12.6±3.32

a: Significantly different with control; b: Significantly different with SMC group; c: Significantly different with HFD+metformin at 5% level of significant (P<0.05)

The details on the comparison of the antioxidant effects of SMC between the study groups are given in table 2. It shows that the parameters such the level of malondialdehyde, Glutathione peroxidase and GSH were significantly different (P<0.05) on overall between the groups. The level of malondialdehyde (4.28) was significantly higher in the HFD group compared with all other groups. The level of Glutathione peroxidase was lower in the HFD group (29.36) compared to all other groups but shows significant difference (P<0.05) only with HFD+metformin group. The level of GSH shows significantly lower (P<0.05) in the HFD group (3.42) compared with all other study groups. However, the level of catalase does not show significant differences (P>0.05) on overall between the groups. However, the pairwise analysis shows that the level of catalase is lower in the HFD group compared with all other groups but the difference was statistically significant (P<0.05) only while comparing with HFD+metformin groups. Treatment with SMC in the dose of 100 mg/kg body weight per day as well as metformin (50 mg/kg body weight per day) significantly decreased the plasma malondialdehyde and increased the whole blood reduced glutathione, erythrocytes GPx and catalase activities.

**Table 2:** Antioxidants effects of SMC in control and experimental groups of rats

**Groups**	**MDA (µM/L)**	**CAT (katal/ml)**	**GPx (mg/g Hb)**	**GSH (mg/g Hb)**
Control	2.56±0.28	42.70±18.43	42.89±8.19	6.57±1.45
Control+SMC	2.68±0.46	41.04±10.78	51.83±13.11	9.64±0.47
HFD	4.28±0.88a,b,c	30.99±4.64c	29.36±5.76c	3.42±0.62a,b,c
HFD+SMC	2.91±0.17	47.54±16.09	43.56±10.12	6.75±1.20
HFD+metformin	2.59±0.30	56.92±16.09	51.69±7.03b	7.38±1.24

a: Significantly different with control; b: Significantly different with SMC group; c: Significantly different with HFD+metformin at 5% level of significant (P<0.05)

**Figure 1:**
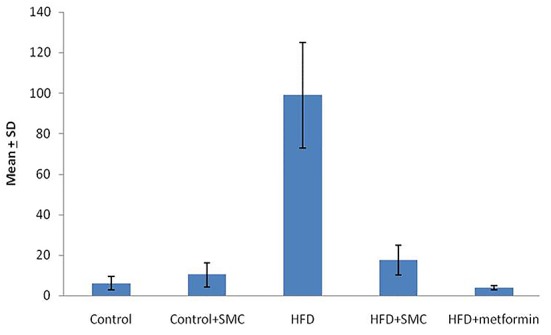
Comparison of the level of TNF-α between the study groups. The TNF-α is significantly higher in HFD when compared with all other groups (P<0.05)

The effect of SMC on the level of TNF-α between the study groups shows that TNF-α is significantly higher (P<0.05) among HFD induced rats when compared with other groups. The details on the analysis of TNF-α between the study groups is depicted in figure 1. A significant (P<0.05) decline in the levels of TNF-α was observed on oral administration of SMC as well as metformin.

## Discussion

Metabolic syndrome is a major health problem worldwide and is associated with complications, such as hypertension, inflammation, hyperinsulinemia, insulin resistance, and cardiovascular disorders. Fructose is widely used as a food ingredient and has the potential to increase oxidative stress and related complications. There are many reports in the literature describing an increase in hyperglycemia, and hyperinsulinemia with the consumption of high fructose diets in both humans and animal models.^16^^,^^17^. Our results are consistent with previous studies, which found that the intake of fructose rich diets markedly induce impairment in glucose tolerance leading to hyperglycemia and hyperinsulinemia.^18^ The major finding of the study was the treatment of SMC that significantly improves the high fructose induced oxidative stress as indicated by the decreased malondialdehyde and increased catalase, GPx and reduced glutathione activities and insulin resistance as indicated by decreased glucose, insulin and HOMA–IR.

A high flux of fructose to the liver, could elicit rapid responses that ultimately influence hepatic gene expression, glucose disposal, and insulin action. This mechanism attributed to the fact that fructose metabolism bypasses the regulatory step catalyzed by phosphofructokinase-1. Thus, fructose continuously enters the glycolytic pathway resulting in lipogenesis.^19^ The excess glucose released into the blood, stimulates more insulin secretion, leading to reduced insulin sensitivity.^20^ In the HFD treated rats, the mechanism for the hypoglycemic effect of SMC is believed to be related to its role in the regulation of glycolytic pathway.

The chronic diabetes could lead to the cell membrane lipid peroxidation due to the formation of highly reactive species. The pathologic complications of membrane lipid peroxidation include the formation of lipid peroxides affect the membrane lipid modification, cellular deformability, and altered membrane fluidity in mainly erythrocytes and other tissues.^21^ The results of the present study are also in line with the previous studies, where SMC supplementation to HFD group of rats reduced level of plasma malondialdehyde.^22^ In addition, compared with SMC the treatment with metformin dramatically reduces the MDA but no statistical significant was found between HFD+SMC and HFD+metformin groups.

Catalase (CAT) is a hemeprotein, which catalyses the reduction of hydrogen peroxides and protects the tissues from oxidative stress. This decrease in CAT activity could result from inactivation by glycation of enzyme.^23^^,^^24^ Reduced activities of catalase in the red blood cells as well as tissues have been found during diabetes and this may result in a number of metabolic derangements due to the accumulation of free radicals such as superoxide radicals and hydrogen peroxides.^25^ Treatment with SMC and metformin reversed the activity and thereby minimizing the effect of stress induced damages but no statistical significant was found between HFD+SMC and HFD+metformin groups.

Glutathione (GSH) is the most abundant thiol antioxidant present in the blood and tissues, together with its associated biosynthetic, redox and detoxification pathways represents the key defense system against oxidative stress and free radical damage in the cell. In addition, GSH plays numerous protective roles including the detoxification of a variety of endogenous and exogenous compounds such as xenobiotics and carcinogens, preservation of protein structure function and synthesis.^26^ A marked decrease in the level of GSH in erythrocyte during diabetes was observed. Various studies support the hypothesis that in type 2 diabetes, chronic hyperglycemia increases the polyol pathway as well as advanced glycation end products (AGE) formation and free radical generation, leading to increased oxidation of reduced glutathione. A relative depletion of NADPH due to aldose reductase activation and secondary to reduced production through the HMP shunt pathway impairs GSH synthesis and leads to decrease of this free radical scavenger.^27^ GPx catalyze the conversion of hydrogen peroxide to water using GSH as a hydrogen donor. The reduced activity of GPx may result in the accumulation of toxic products due to oxidative damage. The significant recovery of GSH content and GSH dependent enzyme GPx by treatment of SMC indicates the antioxidant property of SMC and the latter will potentially protect the cells from free radical damage.

Intake of fructose rich diet is considered as one of the factors that likely contribute to excessive generation of reactive oxygen species. This leads to oxidative stress and its associated complications like chronic inflammation, characterized by abnormal cytokine production (TNF-α) and the activation of a cascade of inflammatory signaling pathways.^28^ TNF-α has been shown to enhance adipocyte lipolysis, which further increases free fatty acids and also elicits its own direct negative effects on the insulin signaling pathway by altering the tyrosine and serine phosphorylation of insulin receptor substrate (IRS).^29^^,^^30^ Our results demonstrate that SMC treatment in HFD rats caused a significant decrease in plasma TNF-α, further supporting its usefulness in the treatment of type 2 diabetes. The same result was also found in the metformin treated groups.

## Conclusion

The present study indicates that, the s-methyl-L-cysteine has potential antioxidant activity and this will enable to prevent or reduced the development of high fructose induced oxidative stress, inflammation, and insulin resistance. Further studies are required at the molecular level to explore the mechanism by which s-methyl-L-cysteine reduces the above-mentioned complications. 
